# The rise and fall of a social support intervention feasibility trial targeting loneliness in patients with cardiac disease - lessons learned and future perspectives

**DOI:** 10.1186/s12912-024-02113-6

**Published:** 2024-06-24

**Authors:** Mitti Blakoe, Cathrine S. Olesen, Anne Vinggaard Christensen, Pernille Palm, Ida Elisabeth Hoejskov, Selina Kikkenborg Berg

**Affiliations:** 1 The Heart Centre, Copenhagen University Hospital, Rigshospitalet, Copenhagen, Denmark; 2 Faculty of Health and Medical Sciences, University of Copenhagen, Copenhagen, Denmark

**Keywords:** Loneliness, Social support, Feasibility, Cardiac, Intervention, Clinical nursing

## Abstract

**Background:**

One of the psychosocial factors recognized for its positive impact on health outcomes among patients with heart disease, is social support provided by network members. However, an increasing number of patients report to experience loneliness. This study addresses the gap in research on the feasibility of an individually structured social support intervention targeting patients treated for cardiac disease who experience loneliness.

**Method:**

A feasibility trial of a 6-month social support intervention targeted patients treated for cardiac disease who experienced loneliness. The intervention involved providing the patient with an informal caregiver, either a person from the patient’s social network or a peer, in the long-term rehabilitation phase. Furthermore, the intervention included nurse consultations and motivational text messages. Feasibility was assessed in terms of acceptability and adherence.

**Results:**

During October 2022-July 2023, *n* = 464 patients were screened for loneliness and 28 (6.0%) screened positive of which 17 (60.7%) accepted to be contacted and receive additional information about the social support intervention. Of these, 2 (11.8%) accepted participation. The low recruitment rate did not meet the predetermined acceptability criterion of 25%.

**Conclusion:**

This individually structured social support intervention targeting patients treated for cardiac disease who experience loneliness was non-feasible. The study highlights the complexities of engaging lonely patients in a social support intervention program and contributes with valuable insights for future research aiming to develop effective social support interventions tailored to the needs of cardiac patients who experience loneliness.

**Trial registration:**

The trial is registered on clinicaltrials.gov (NCT05503810) 18.08.2022.

## Background

Despite advanced medical treatments and improved disease prevention, it is estimated that more than half a billion people are living with heart disease worldwide [1, 2]. One of the psychosocial factors recognized for its positive impact on health outcomes among people with heart disease, is social support provided by network members [3–5]. Evidence suggests that social support has the potential to function as a protective factor against the progression and complications of heart disease by e.g., reducing stress levels, increasing compliance with treatment regimes [6, 7] and promote healthy behaviors [8]. Beyond the influence of social support on health outcomes, social support is crucial in helping patients manage the physical and psychological aftermath of in-hospital cardiac treatments [9, 10].

However, national health surveys suggest that the number of people who reports experiencing loneliness is increasing [11]. Loneliness can be defined as follows: “A distressing feeling that accompanies the perception that one’s social needs are not being met by the quantity or especially the quality of one’s social relationships” [12]. In patients treated for cardiac disease, it is reported that 4% repors loneliness [13] and that loneliness is significantly associated with an increases risk of one year mortality [14]. In sum, patients who experiences loneliness are placed in a vulnerable situation during admission and in the rehabilitation phase.

While the importance of social support for patients with cardiac disease is widely recognized, there remains a need for research to enhance the development of social support intervention programs targeting patients who experience loneliness, as also recommended by in a Scientific Statement from the American Heart Association [15] and by the European guidelines for cardiovascular disease prevention [16].

Social support interventions can take various structures i.e., it may involve informal caregivers from the patient’s existing social network, or it may involve peer support (someone with the same lived experience as the recipient) or it may have components of cognitive behavioral therapy, motivational text messaging, or mindfulness. Social support interventions targeting cardiac disease patients in general, (i.e., whether they feel lonely or not), suggest that interventions involving informal caregivers from the patients’ existing social network may offer a promising avenue for enhancing social support [17] and may have a small too modest effect on blood pressure, risk of hospital admission and overall quality of life [18]. Similarly, peer support interventions have demonstrated some positive effects on improved activity, reduced pain, fewer emergency room visits, and increased cardiac rehabilitation attendance [19, 20]. One theory posited to promote the positive impact of social support on health behavior is the Middle-Range Theory on Self-care [21]. According to this theory, self-care has three interrelated components: maintenance, monitoring, and management, all of which can be facilitated by social support. To illustrate, social support can increase the motivation to make healthier choices, it can support the patients in performing adequate monitoring and disease management or it may help the patient to remember and understand the advice of health professionals, and, to transfer the advice into the patient’s everyday life [21, 22].

However, research targeting patients who experience loneliness is sparse and therefore, the evidence of which structures are suitable for this sub-population is lacking. In previous research, patient involvement sessions involving patients treated for cardiac disease who experience loneliness has illuminated that a social support intervention must align with individual patient preferences e.g., the type of informal caregiver (peer vs. network member), and intervention frequency and duration, to be an appealing proposition. Further details on insight from the patient involvement sessions is described elsewhere [23]. Based on insights from these patient involvement sessions and insight from existing literature regarding social support interventions, we composed an intervention targeting patients treated for cardiac disease who experience loneliness.

Given the absence of prior studies investigating individually tailored social support interventions in this sub-patient population, the study was conducted as a feasibility trial.

### Study aims and objectives

The primary aim of the study was to determine the feasibility of an individually structured social support intervention targeting patients treated for cardiac disease who experience loneliness. The secondary aim was to explore the preliminary evidence on the effect of the intervention on health behaviors and health outcomes.

This study adheres to CONSORT 2010 guidelines: extension to randomized pilot and feasibility trials [24].

## Methods

### Trial design

This feasibility study was conducted in a randomized clinical trial (RCT) design as described in detail elsewhere [25]. In short, the RCT design entailed a 1:1 randomization approach allocating patients into two groups: one group receiving a 6-month social support intervention in addition to usual follow-up (intervention group), and the other group receiving usual follow-up (control group) as described in the National Treatment Guidelines [26]. The study aimed to involve 20 patients in each group.

### Participants

Patients from three treatment groups: (i) Surgical procedures (coronary artery bypass grafting (CABG), surgical aortic valve replacement (SAVR), surgical mitral valve procedures, (ii) non-surgical, invasive procedure (percutaneous coronary intervention (PCI), implantable cardioverter defibrillator (ICD), pacemaker implantation, ablation) or (iii) medical treatment, treated at Copenhagen university hospital were approached. Initially, convenience sampling was utilized to recruit participants. Patients were approached face-to-face during admission (*n* = 184), by an experienced project nurse, or by first author. To optimize the recruitment rate, we converted to consecutive sampling where patients were approached within one week of discharge (*n* = 564) through the digital platform ‘Digital Post’ (a secure digital postbox used by the Nordic authorities to communicate with citizens. Approximately 93% of the Danish population uses ‘Digital Post’) to be answered in REDCap [24] (a web application for online surveys). Patients were excluded if they were unable to provide written consent, therefore, patients with severe cognitive or physical dysfunction were not approached.

At the initial approach, patients were screened for loneliness with the HiRL screening tool [27]. Classification of loneliness is described in Table 1.

**Table 1:** High-Risk Loneliness (HiRL) screening tool

Screening question	Answer	Point	Classification of high-risk loneliness
*“Does it ever happen that you are alone even though you wish to be with others?”*	No		≥ 1 point
	Yes, but rarely		
	Yes, sometimes		
	Yes, often	1	
*“Do you have someone to talk to if you experience problems or need support?”*	Yes, often		
	Yes, most of the time		
	Yes, sometimes		
	No, never or rarely	1	

Patients who screened positive for loneliness were provided with oral and written information about the study and asked for permission to be contacted and receive information about the social support intervention study. Patients who agreed to be contacted received further oral and written information about the social support intervention program and if acceptance was achieved, they signed informed content before randomization. Randomization was completed using the web-based tool Redcap. Stratification was based on sex (male/female).

### Intervention

The core content of the intervention was to provide the patient with an informal caregiver in the long-term (six-month) rehabilitation phase following cardiac disease treatment. As the intervention was based on an individual structure, the informal caregiver could either manifest as a network member (e.g., a partner, friend, or neighbor), formally assuming the role of an informal caregiver, or as a peer, depending on the patient’s preference. Peers were recruited from the Danish Heart Foundation among volunteers in an existing peer support program, where they have received structured training in e.g., conversation techniques and in providing emotional support to people with critical illness.

After selecting their preferred type of informal caregiver, patients were queried regarding the frequency and method of interaction with the informal caregiver. It was recommended that the patient and the informal caregiver engage in contact, either in person or remotely (via phone or virtual means), at least once a week. Additionally, patients who owned a mobile phone received motivational text messages, formulated by the research team, with the intent of bolstering the supportive environment [28]. These text messages were automatically dispatched by RedCap to the patient on Mondays between 1:00 and 3:00 PM.

The primary focus of the intervention was to enhance and reinforce the informal caregiver’s competencies to serve as a social support resource through nurse consultations. This central component remained consistent, regardless of whether the patient selected an informal caregiver from their social network or selected a peer. Upon enrollment, at one month, and three months into the intervention, an intervention nurse established communication with the informal caregiver and offered guidance and counseling as outlined in a specific theoretical framework, described elsewhere [25]. In short, Middle-Range Theory on Self-care [21] was chosen as a theoretical framework in the consultations. The use of this framework has the potential to inform the consultation nurse on which supporting actions to promote to the informal caregiver, depending on where in the self-care process the patient is struggling, i.e. maintenance, monitoring, or management. Both the patient and the informal caregiver were provided with the option of reaching out to an intervention nurse via an open hotline during working hours if any additional queries appeared throughout the intervention period.

## Outcome measures

### Feasibility

Feasibility was evaluated in terms of acceptability and adherence [29, 30]. Before inclusion of the first patient feasibility criteria were determined. Acceptability in patients was supported if 25% of ptients screened as lonely agreed to participate in the trial. We chose this recruitment rate as the literature indicates that vulnerable patients can be difficult to recruit [31] and it therefore seemed to be a realistic rate. Acceptability in social network members was supported if 50% of invitd social network members accepted participation. Adherence in patients was supported if 75% adhered o the intervention i.e., had contact with the informal caregiver once a week for a minimum of 8 out of 12 weeks. Adherence in informal caregivers (social network members or peer) was supported if 75% participted in two out of three nurse consultations.

Additionally, in feasibility and pilot testing it is recommended to monitor the resources used to complete the study [30]. Thus, time resources used for inclusion and nurse consultations were recorded as described in detail in the study protocol [25].

### Health behaviors

At baseline, demographic characteristics (age, sex) and treatment group was obtained from medical records. At baseline, one-, three-, six-, and twelve-month patients received a questionnaire in `Digital post´ consisting of questions related to smoking, alcohol consumption, height, weight, physical activity, participation in cardiac rehabilitation and readmission, along with the following questionnaires:

### High risk loneliness tool (HiRL)

HiRL is a two-item social support questionnaire that originates from the Danish National Health Survey [32]. Respondents can answer on a 4-point Likert scale ranging from 1 ‘Yes, often to’, to 4 ‘No, never’, with higher scores indicating greater loneliness. The questionnaire has been demonstrated to have a predictive value of one-year mortality with a sensitivity of 19.9% and specificity of 89.5% [27].

#### Self care self-efficacy scale (SCSES)

SCSES is a 10-item questionnaire that measures self-efficacy related to self-care maintenance, monitoring, and management in patients with chronic illness. Respondents can answer on a 5-point Likert scale [33].

#### Health-related quality of life (HeartQoL)

HeartQoL, is a 14-item heart disease-specific quality of life questionnaire answered on a four-point Likert scale. The questionnaire has been translated and validated in several languages [34].

#### Hospital anxiety and depression scale (HADS)

HADS is a 14-item questionnaire, divided into a seven-item depression subscale and a seven-item anxiety subscale. Respondents are asked to report on how they have felt in the past week. The HADS has been shown to detect depression and anxiety with high sensitivity in cardiac patients [35].

The trial is registered on clinicaltrials.gov (NCT05503810) 18.08.2022.

## Results

During a 10-month period (October 2022-July 2023) a total of 748 patients were screened for high-risk loneliness with the HiRL screening tool (*n* = 184 face-to-face during admission and *n* = 564 patients within one week of discharge through the digital platform). A total of 464 (62.0%) responded to the questionnaire. Of these 28 (6.0%) screened positive for high-risk loneliness and of which *n* = 17 (60.7%) accepted to be contacted by phone and receive additional information about the social support program.

The study achieved a recruitment rate of 23.5% (*n* = 4). Of the recruited patients, three were randomized to the control group and one was randomized into the intervention group. Of these, two (one in the intervention group and one in control group) did not reply to further contact following acceptance and were there for deregistered, leaving 11.8% (*n* = 2) of eligible patients in the social support intervention program. Consequently, the feasibility criteria on acceptability of 25% was not supported. As the two patients who accepted participation were randomized in the control group neither adherence to the intervention nor efficacy could be monitored. And overview of the screening and recruitment process is illustrated in Flowchart 1.

**Flowchart 1:**
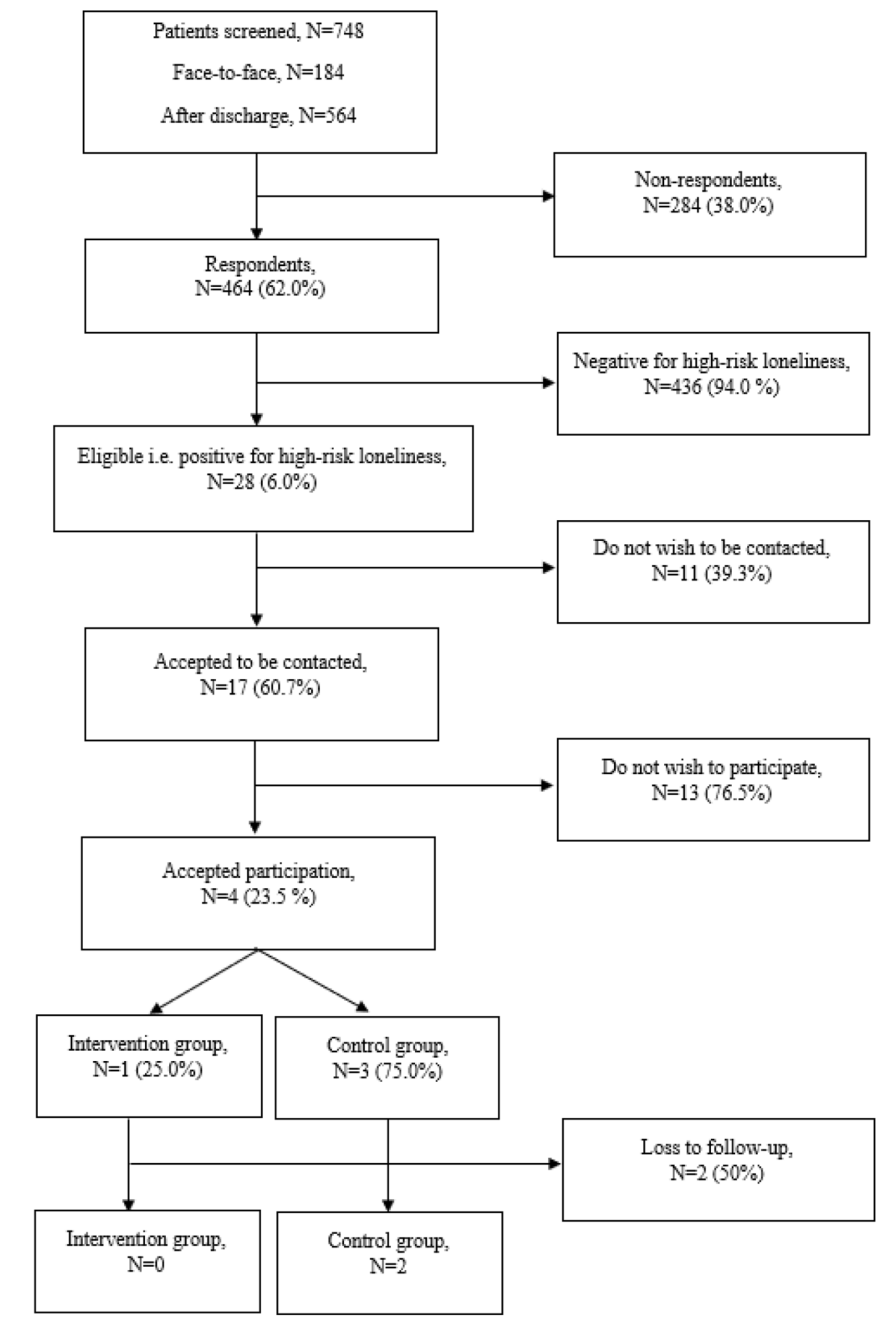
Screening and recruitment process

## Discussion

To the best of our knowledge this is the first study to investigate the feasibility of an individually tailored social support intervention, targeting patients with cardiac disease, who experience loneliness.

Despite that the social support intervention was based on insights from patient involvement sessions and existing social support theory, the study revealed a low acceptance rate and thus, the program was deemed to be non-feasible. Based on the verbal responses from the invited patients who did not accept participation, and drawing on theory in the field, valuable insights are gained regarding the potential barriers in this specific subpopulation to engage in a social support intervention.

One potential barrier may be timing of recruitment of participants. Initially, we approached patients during hospitalization, informing them about the study if they were identified as lonely with the HiRL tool [27]. However, we learned that few patients possessed the mental capacity to consider participation during admission. Consequently, we adapted our approach and opted to distribute the questionnaire digitally within a week post-discharge. The decision to administer it shortly after discharge was informed by insights gained from patient involvement sessions, wherein participants expressed a particular need for social support in the early post-discharge period [23]. In addition, provision of support in the early post-discharge period may help patients to understand their illness and adapt to a healthier lifestyle [36]. Nevertheless, the modified recruitment timing did not alter the acceptance rate. This observation is likely rooted in the understanding that the initial days following discharge constitute a stressful period wherein, despite the substantial need for social support [7], patients may find it overwhelming to engage in new social relationships. This finding is also reflected in theories regarding social isolation among individuals with chronic illness, which describe that higher levels of chronic illness intrusiveness impede social participation [37].

Another potential barrier may arise from the requirement for patients to complete an extensive questionnaire as part of their participation in the study—a process that can be daunting, especially during a stressful period. This challenge may be particularly pronounced among patients experiencing loneliness, as this sub population, akin to other vulnerable patient groups, has exhibited a tendency towards lower rates of participation in health-related research [38]. This experience holds a significant value, as it indicates the importance of being especially attentive to utilizing brief questionnaires when working with vulnerable groups, as also highlighted by Bonevski, B, et al. [31]. In the context of a potential future study on a social support intervention targeting patients who experience loneliness, this can be affected by exclusively prioritizing the feasibility outcome measure rather than concurrently focusing on heath related outcome measures.

Finally, even though the intervention was designed to take the individual participants’ preferences regarding type of informal caregiver (peer vs. network member) into consideration as recommended in the literature [17, 23, 39], patients may have several reasons for opting out of social relationships. To illustrate, health psychologists suggest that people who feel lonely tends to see the social world as a more threatening place and to have more negative social expectations [12], which may explain why many of those invited were reluctant to accept participation. On the other hand, evidence points to that social support is needed during admission and in the early rehabilitation period [9]. In sum, future research advantageously delves deeper into determining the optimal structure and focus for a social support intervention to make it acceptable for patients experiencing loneliness.

### Strengths and limitations

A strength of this study is that the social support intervention design was informed by insights obtained from patient involvement sessions within the target population. This approach was pertinent given the limited existing literature on social support interventions specifically for patients facing loneliness.

Given that loneliness can be experienced as a stigma, there is a possibility of introducing selection bias during the recruitment process, wherein some individuals may opt out of participation. Additionally, the introduction of desirability bias is conceivable, as respondents may provide answers incongruent with their actual experiences of social support. Selection bias might also have arisen in the digital distribution of questionnaires, as this method could serve as a barrier for certain patients.

The decision to use a 2-item questionnaire for identifying patients experiencing loneliness was predicated upon a desire to distribute an easy-to-use instrument that imposed minimal burden on respondents. This choice likely contributed, on one hand, to the high response rate of the questionnaire (62%), however, on the other hand, it may have introduced inaccuracies or resulted in low sensitivity in detecting patients in need of social support.

## Conclusion

This individually tailored social support intervention, providing patients who experience loneliness with an informal caregiver in the long-term (six-month) rehabilitation phase following cardiac disease treatment, was non-feasible.

The study highlights the complexities of engaging patients who experience loneliness in social support intervention programs. Further research is needed to explore alternative recruitment strategies and refine intervention structures to enhance acceptability and feasibility in this vulnerable patient population. This feasibility trial contributes valuable insights for future research aiming to develop effective social support interventions tailored to the needs of patients treated for cardiac disease who experience loneliness.

## Data Availability

The datasets used and/or analysed during the current study are available from the corresponding author on reasonable request.
